# CYP116B5hd, a self-sufficient P450 cytochrome: A dataset of its electronic and geometrical properties

**DOI:** 10.1016/j.dib.2022.108195

**Published:** 2022-04-25

**Authors:** Antonino Famulari, Danilo Correddu, Giovanna Di Nardo, Gianfranco Gilardi, Mario Chiesa, Inés García-Rubio

**Affiliations:** a Department of Condensed Matter Physics, Faculty of Sciences, University of Zaragoza, Calle Pedro Cerbuna 12, Zaragoza 50009, Spain; b Department of Chemistry, University of Turin, Via Giuria 9, Torino 10125, Italy; c Department of Life Sciences and Systems Biology, University of Turin, Via Accademia Albertina, 13, Turin, 10123, Italy; d Centro Universitario de la Defensa, Carretera de Huesca s/n, Zaragoza 50090, Spain

**Keywords:** Cytochromes P450, EPR, EPR simulations, Ferric hemeprotein, Spin density, HYSCORE

## Abstract

This paper documents the dataset obtained from the Electron Paramagnetic Resonance (EPR) study of the electronic properties of a self-sufficient cytochrome P450, CYP116B5hd, which possesses an interesting catalytic activity for synthetic purposes. In fact, when isolated, its heme domain can act as a peroxygenase on different substrates of biotechnological interest.

Raw data shown in Famulari et al. (2022) and supplementary data in raw and processed forms (figures) are documented and available in this paper. Additionally, simulations of the experimental data together with simulation scripts based for EasySpin, a widespread MATLAB toolbox for EPR spectral simulations, are provided. The procedure for g-value analysis based on a crystal-field theory is also detailed here, offering an interesting tool for comparison of Fe^III^-heme P450 systems.

Due to the catalytic interest of the protein, which has been recently discovered, and the correlation that has been reported between g-values and peroxidase function, both, CW-EPR and HYSCORE spectra and data set of the model CYPBM3hd are also provided.

Finally, the materials and methods for enzyme production and purification, sample preparation and experimental and spectroscopic procedures a together with instrumental details are described in detail.

The data files and simulation scripts can be found in: https://doi.org/10.5281/zenodo.6418626

**Specifications Table**

**Table:** 

Subject	Inorganic Chemistry and Biophysics
Specific subject area	EPR spectroscopy; Cytochromes P450
Type of data	Primary data, Tables, Scripts and Figures
How the data were acquired	The data were collected at low-temperature (6-40 K) using a Bruker Elexys E580 X-band spectrometer (microwave frequency 9.68 GHz) equipped with a cylindrical dielectric cavity and a helium gas-flow cryostat from Oxford Inc.
Data format	Data are in .DTA (file containing the spectral data), .DSC (text file containing the spectral parameters) and .txt (text file containing the spectral data)
Description of data collection	The data were collected as described in Section 5. The spectra obtained were processed with the EasySpin toolbox for MATLAB.
Data source location	Institution: University of Zaragoza and Turin; City/Town/Region: Zaragoza (Aragon) and Turin (Pidmont); Country: Spain and Italy Latitude and longitude (and GPS coordinates, if possible) for collected samples/data: 41.642149°, -0.899461° (Zaragoza) and 45.0498279°, 7.6794686° (Turin);
Data accessibility	Repository name: Zenodo Direct URL to data: *https://doi.org/10.5281/zenodo.6418626*
Related research article	A. Famulari, D. Correddu, G. Di Nardo, G. Gilardi, M. Chiesa and I. García-Rubio, EPR Characterization of the Heme Domain of a Self-Sufficient Cytochrome P450 (CYP116B5), J. Inorg. Biochem. 231 (2022) 111785. https://doi.org/10.1016/J.JINORGBIO.2022.111785.

**Value of the Data**

• The dataset provided consist of a collection of CW-EPR and HYSCORE spectra of CYP116B5hd and CYPBM3hd. Due to the sensitivity of EPR to the close environment of the iron, the availability of such data will be of use to EPR researchers for comparison with future studies of other proteins of the family.
• The simulation scripts will help other heme-protein researchers to get familiar with EPR simulations using EasySpin.
• The detailed explanation of the model for g-value analysis allows to understand the significance and interpretation of the two crystal-field parameters (Δ and V), providing structural insights of the heme site and a way for classification and quantitative comparison between CYP450 enzymes.
• The details about the materials and methods will be of interest for sample preparation of similar protein type or experimental reproducibility.

## 1 Data Description

Data were collected using an EPR spectrometer and processed with EasySpin, a widespread MATLAB toolbox. The dataset in the Zenodo repository (https://doi.org/10.5281/zenodo.6418626) contains raw and processed numerical data. Additionally, below we present figures and tables complementing the ones shown in [1].

*Description of the dataset:*

• **Data type**: Experimental spectroscopic measurements (EPR), computer simulations and data analysis.
• Files are with **filename extensions**: .DSC, .DTA, .txt, and .m
• Information on **origin of the data**:
  ■ EPR spectroscopic measurements have filename extensions .DSC, .DTA and .txt.
  ■ EPR spectroscopic simulation and analyses with filename extension .m.
• **Simulations** of EPR spectra and fitting were performed with the Easyspin software (v. 6.0.0-dev.30) implemented in Matlab (MathWorks, R2021a).
• The **dataset**:
  ■ Files in the folder **PARACAT_WP5_20220225_CW** contain CW-EPR spectroscopic measurements, original data are in .DSC, .DTA and .txt format.
  ■ Files in the folder **PARACAT_WP5_20220225_HYSCORE** contain HYSCORE EPR spectroscopic measurements, original data are in .DSC, .DTA and .txt formats.
  ■ Files in the folder **PARACAT_WP5_20220225_MATLAB** contain: ready-to-plot / ready-to-simulate data in .m format.

## 2 Experimental Design, Materials and Methods

*Cloning, expression and purification of CYP116B5hd***.** The construct used in this work was obtained by cloning the initial part of the gene of CYP116B5 (coding for the first 442 amino acids, the heme domain) between *NdeI* and *EcoRI* restriction sites in a pET-30a (+) vector with the insertion of a N-terminal 6xHis tag [8]. Expression and purification of the protein were carried out as described before [8]. Briefly, protein expression was carried out in *E. coli* BL21 (DE3) cells at 22–24 °C for 24 h in LB medium supplemented with 0.5 mM of δ-aminolevulinic acid (δ-Ala) and 100 μM IPTG. For the purification, cells were resuspended and sonicated in a 50 mM KPi pH 6.8 buffer supplemented with 100 mM KCl, 1 mg/mL lysozyme, 1% Triton X-100 and 1 mM PMSF (phenylmethylsulfonyl fluoride), and 1 mM benzamidine. After ultracentrifugation at 90,000 g for 45 min at 4 °C, the supernatant was loaded onto a 1 ml His-trap HP column (GE Healthcare) and eluted by a linear gradient of imidazole ranging from 20 to 200 mM. The purest fractions were then concentrated, loaded into a Superdex 200 size exclusion chromatography column (GE Healthcare) and eluted using a 50 mM KPi pH 6.8 buffer containing 200 mM KCl. The purified protein was then concentrated and stored in 50 mM KPi pH 6.8 containing 10% of glycerol after buffer exchange by ultrafiltration using Amicon Ultra 30,000 MWCO devices. Protein concentration was estimated from the spectrum of the P450−CO complex upon reduction with sodium dithionite and CO bubbling, using an extinction coefficient of 91,000 M^−1^ cm^−1^ [9].

*Electron Paramagnetic Resonance (EPR)* All the protein samples in buffer KPi 50 mM pH 6.8 10% glycerol were mixed with 30% of glycerol as glassing agent to an approximate final protein concentration of 200 μM. The samples with histidine and imidazole were prepared adding an excess of these chemicals to reach the ratio (1:10) with respect to the protein. The experiments were performed on a Bruker Elexys E580 X-band spectrometer (microwave frequency 9.68 GHz) equipped with a cylindrical dielectric cavity and a helium gas-flow cryostat from Oxford Inc. The samples were and kept continuously frozen with liquid nitrogen to preserve the integrity of the protein

*Continuous-Wave EPR.* The spectra were recorded at 20 K or 40 K and a microwave power of 0.07 mW, modulation amplitude of 1 mT and a modulation frequency of 100 KHz were used. The spectra were baseline corrected and smoothed then simulated by using the EasySpin toolbox for MATLAB [10].

*Hyperfine Sublevel Correlation (HYSCORE)*[11]*.* Pulse EPR experiments were performed at 6 K using the pulse sequence π/2 – *τ –* π/2 – *τ*_1_ – π – π_2_ – π/2 – *τ –* echo with microwave pulse lengths *τ*_π/2_ = 16 ns and *τ*_π_ = 16 ns. The time intervals *t*_1_ and *t*_2_ were varied in steps of 16 ns or 24 ns. *τ* values of 208 ns or 250 ns were chosen. A four-step phase cycle was used for eliminating unwanted echoes. The time traces of the HYSCORE spectra were baseline corrected with a third-order polynomial, apodized with a Hamming window and zero filled. After two-dimensional Fourier transformation, the absolute value spectra were calculated.

## 3 CW-EPR and HYSCORE Spectra of CYPBM3hd

**Fig. 1:**
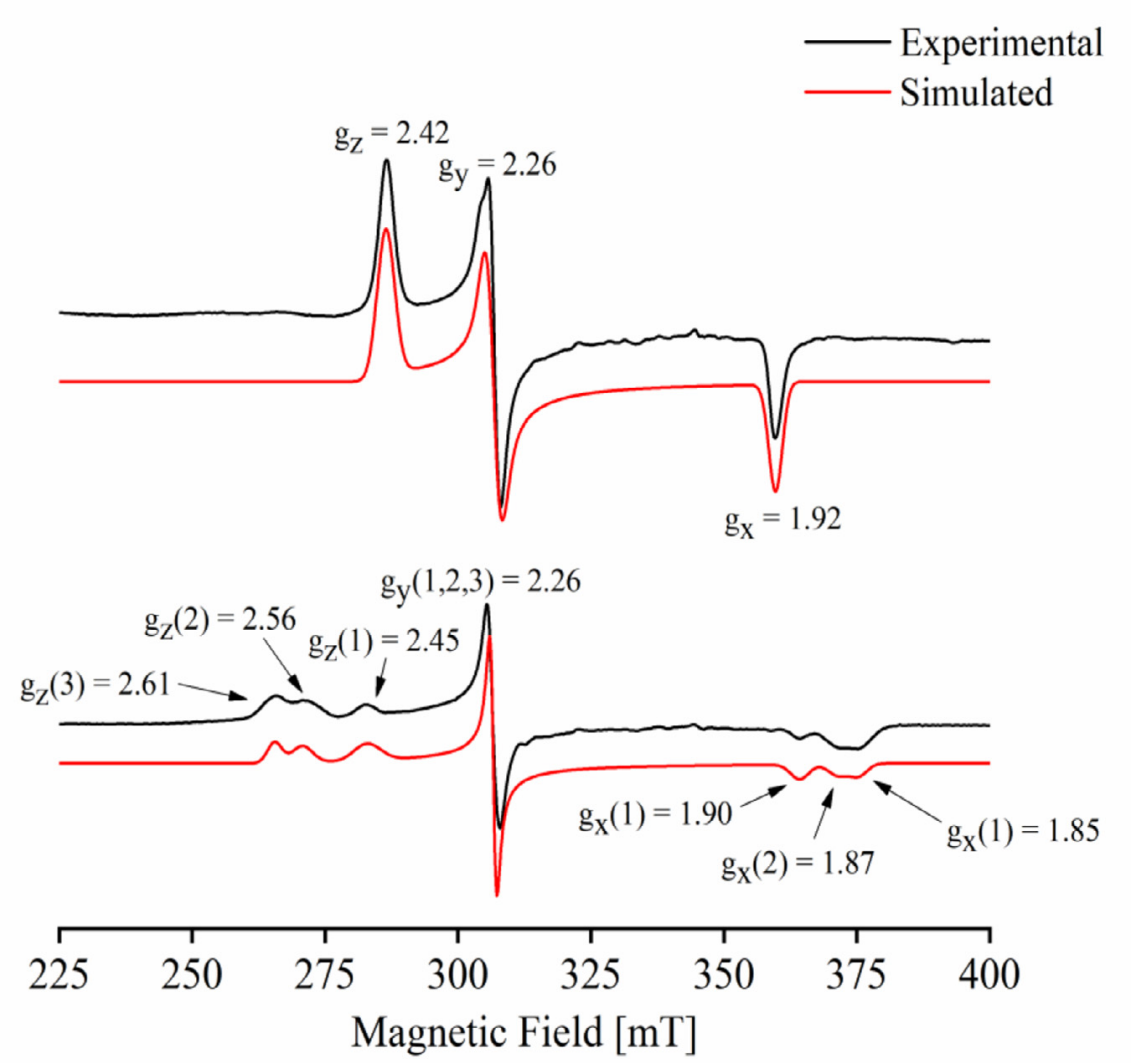
Experimental (black line) and simulated (red line) X-band CW EPR spectra of a frozen solution of (a) CYPBM3hd, in the resting state and (b) CYPBM3hd upon an addition of an excess of imidazole. Buffer is 30 mM KPi pH 7.4, 37% glycerol. Both spectra were recorded at *T* = 20 K.

For comparison purposes, analogous experiments to the ones showed in [1] were performed on the model self-sufficient CYPBM3hd, as shown in Fig. 1.

**Fig. 2:**
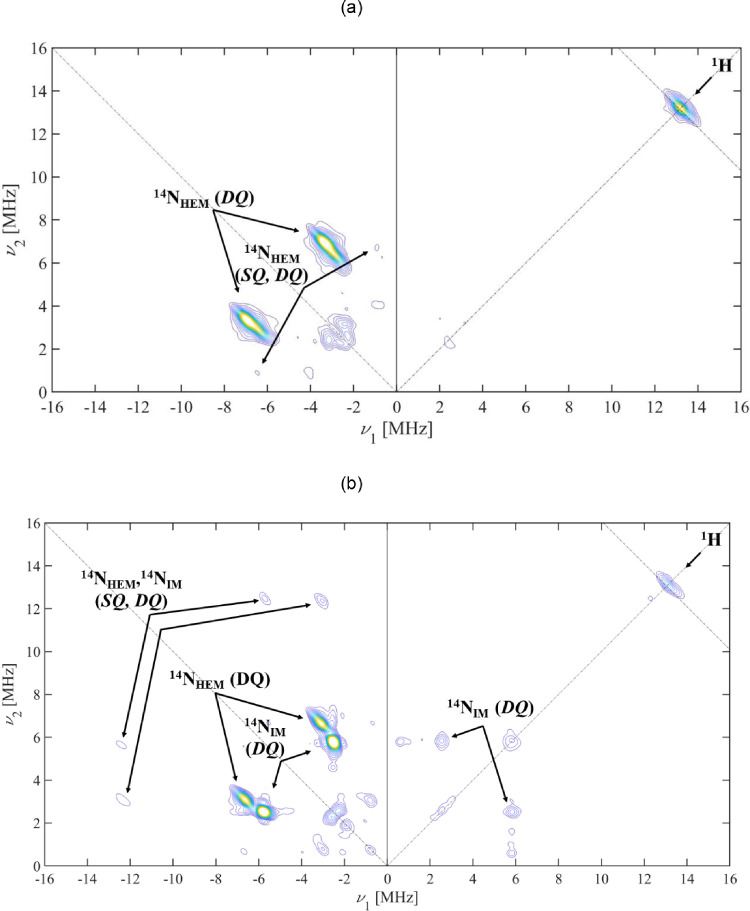
HYSCORE spectra of frozen solutions of (a) CYPBM3hd recorded at the field position corresponding to the *g_y_* feature (*B* = 308.8 mT), witha*t* = 208 ns and (b) CYPBM3hd with an excess of imidazole recorded at the field position corresponding to the *g_y_* feature (*B* = 306.4 mT), with a*t* = 208 ns. Both spectra were recorded at *T* = 10 K. N_HEM_ and N_IM_ indicate the heme and imidazole nitrogens respectively.

Supporting the CW-EPR spectra, the appearance of extra peaks in Fig. 2b, due to an additional strongly coupled nitrogen, indicates that imidazole also displaces the axial water in CYPBM3hd.

## 4 Simulation Parameters of CW-EPR Spectra

The parameters used for simulating the CW-EPR spectra shown in Fig. 1 of [1] are collected in Table 1.

**Table 1:** Parameters calculated from the simulation, performed with the EasySpin toolbox for MATLAB, of all the CW-EPR spectrum showed here and in [1].

Spectrum	g_z_	g_y_	g_x_	g-Strain	Linewidth
CYP116B5hd	2.44 ± 0.005	2.25 ± 0.002	1.92 ± 0.002	0.040, 0.0080, 0.010	2 mT
CYP116B5hd + Imidazole	2.47 ± 0.005	2.26 ± 0.002	1.90 ± 0.002	0.042, 0.0099, 0.020	1 mT
CYP116B5hd + Histidine	2.42 ± 0.005	2.24 ± 0.002	1.92 ± 0.002	0.038, 0.0140, 0.017	2 mT
CYPBM3hd	2.42 ± 0.005	2.26 ± 0.002	1.92 ± 0.002	0.020, 0.0001, 0.001	3 mT
CYPBM3hd + Imidazole	2.45 ± 0.005	2.26 ± 0.002	1.90 ± 0.002	0.050, 0.0005, 0.020	1 mT

## 5 Simulation of CYP116B5hd ^1^H HYSCORE Spectrum

**Fig. 3:**
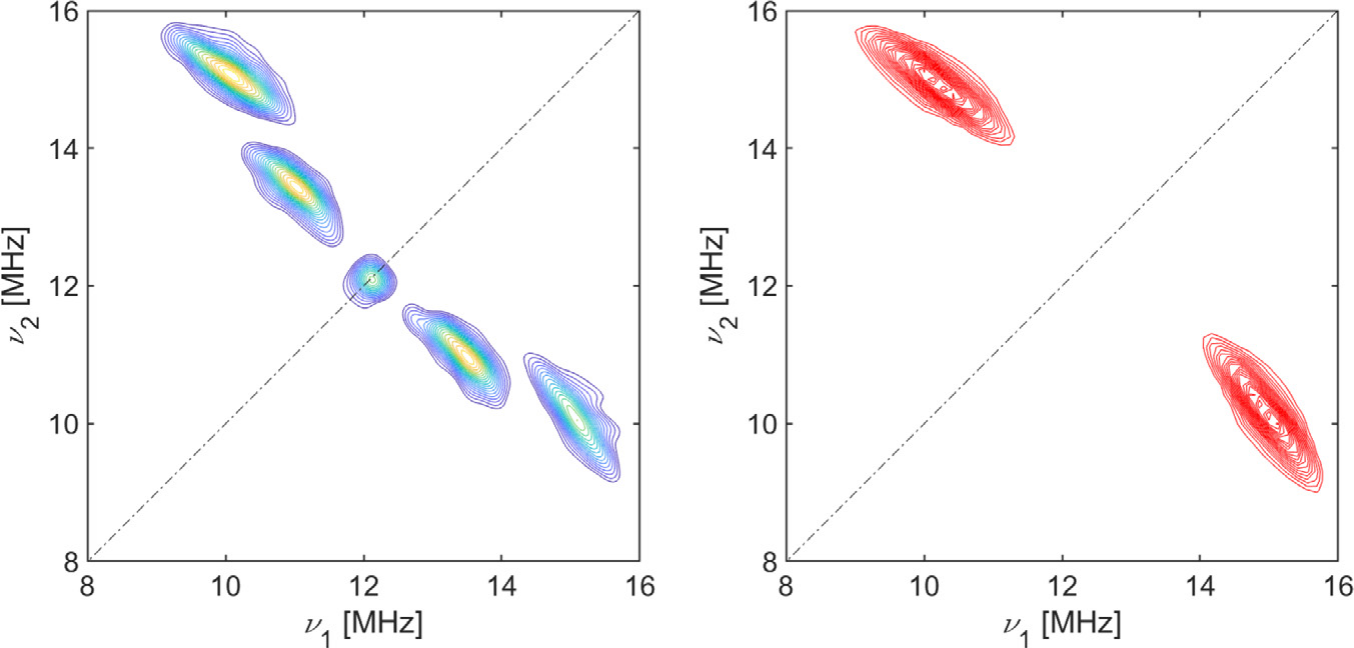
Experimental (left) and simulated proton signal (right) X-band HYSCORE spectra of a frozen solution of CYP116B5hd recorded at the field position corresponding to the *g_z_* feature (*B* = 283.8 mT), witha*t* = 250 ns, at T = 6 K.

The simulation of the experimental HYSCORE spectrum (Fig. 3) allowed to obtain the hyperfine coupling parameters, *a_iso_* = -1.095 MHz and *T* = 5.20 MHz with a b angle of 22°. The dipolar hyperfine coupling value, *T*, can be utilized to determine the radial distance, *r*, between the electron spin and the coupled proton through the point-dipole approximation: $T=\frac{\mu }{4\pi h}\frac{g_{e}\beta_{e}g_{n}\beta_{n}}{r^{3}}$. The formula gave a radial distance value of 2.48 Å.

## 6 Analysis of Low-spin Fe^III^ EPR Signals

One of the most useful approaches when interpreting the *g*-values of a heme low-spin Fe^III^, a d^5^ species, is to consider the relationship between its electron configuration and the gyromagnetic tensor (*g*). The orientation of the tensor is explained by the so-called counter-rotation theory, considered in detail somewhere else [2,3].

We will discuss in the following the connection between the principal values of the tensor and the composition of the ground state.

**Fig. 4:**
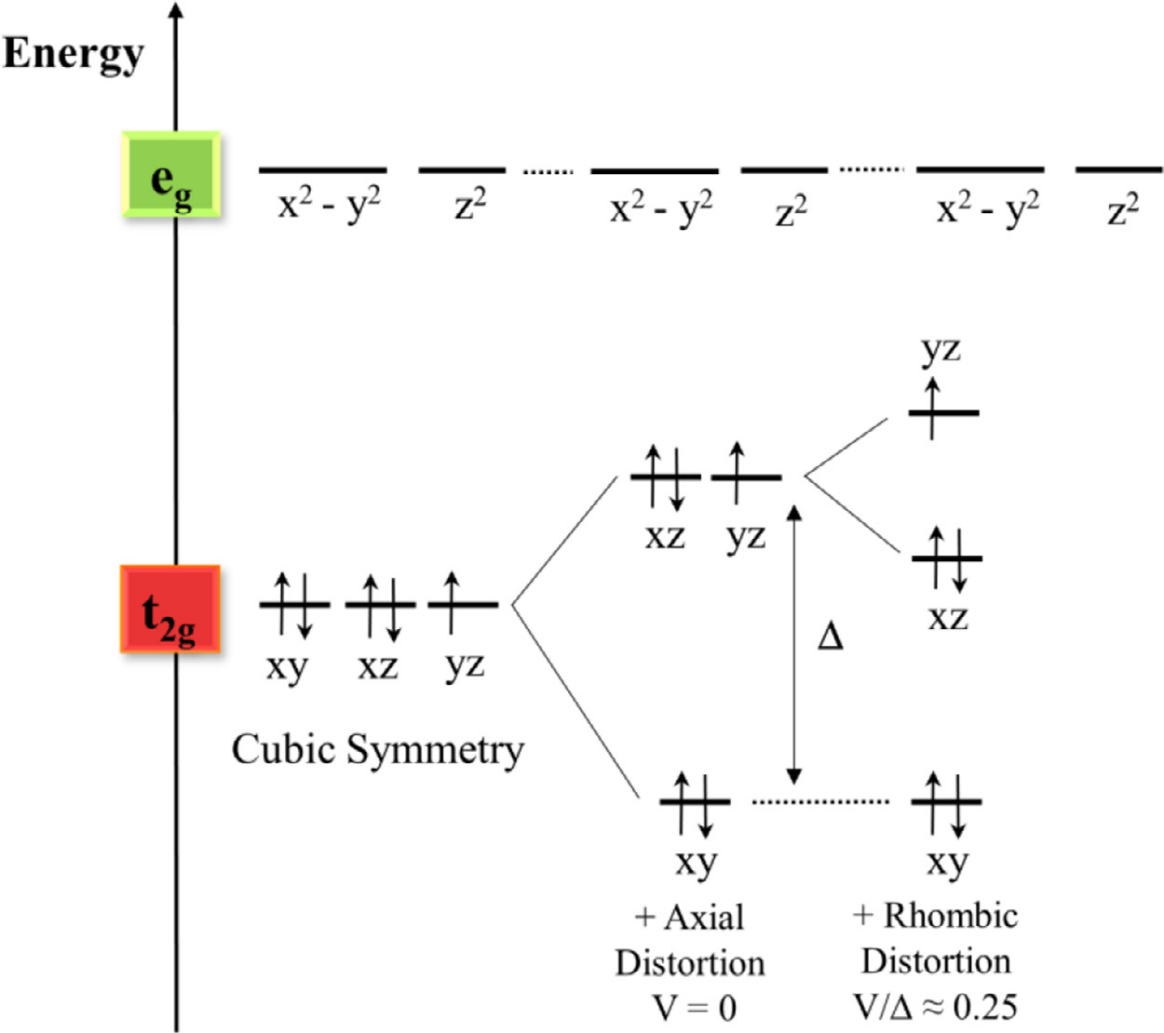
Schematic representation of low spin Fe^III^ d orbitals and their distortion following interaction with a ligand.

The energy levels of the *d* orbitals are defined by the metal surroundings: the nature of the ligands and their geometry. Provided approximate octahedral symmetry and strong ligands, all five *d* electrons of the metal are located in the *t_2g_* orbitals, *d*_xy_, *d*_xz_ and *d*_yz_, resulting in a total electron spin *S* = ½ (low-spin). The energy distribution of the *t_2g_* orbitals can be expressed in terms of the rhombic (*V*) and axial (Δ) crystal field parameters, as schematized in Fig. 4. The difference in the ligand-field strengths between axial ligands and N-porphyrin ligands induces the splitting in energy between *d*_xy_, and the other two *d_xz_* and *d_yz_*, parametrized by Δ. In turn, asymmetries in the axial ligands can split the energy of *d_xz_* and *d_yz_*_._, *V* represents this energy difference. Therefore, Δ is sensitive to the presence and nature of axial ligands and to the strength of their interaction with the metal whereas *V* is related to the nature of the axial ligands and its orientation with respect the porphyrin ring.

According to the formalism introduced by Griffith [4] and developed by Taylor [5], this low-spin system can be treated as a monoelectronic system (one hole) where the spin-orbit coupling energy is of the same order of magnitude than the described effects of the ligands. This results in a ground state that can be described as an admixture of the former three *t_2g_* orbitals via spin-orbit coupling.

Following this model, the wavefunctions for the Kramer's ground doublet are given by Eqs. (1) and (2), where *a, b*, and *c* are orbital coefficients:

(1) $$\left|+\rangle \;=\;a\right|d_{\text{yz}}^{+}\rangle \;-\;\text{ib}|d_{\text{xz}}^{+}\rangle \;-\;c|d_{\text{xy}}^{-}\rangle$$

(2) $$\left|-\rangle =\;-a\right|d_{\text{yz}}^{-}\rangle \;-\;\text{ib}|d_{\text{xz}}^{-}\rangle \;-\;c|d_{\text{xy}}^{+}\rangle$$

The *g*-tensor principal values can be expressed in terms of the mixing coefficients *a, b*, and *c* through the following expressions:

(3) $$\begin{array}{c} g_{z}=2\left[{\left(a+b\right)}^{2}-\;c^{2}\right]\\ g_{y}=2\left[{\left(a+c\right)}^{2}-\;b^{2}\right]\;\\ g_{x}=2\left[a^{2}-{\left(b+c\right)}^{2}\right] \end{array}$$

Conversely, when the g-values are known from experiment, the coefficients can be calculated:

(4) $$a\;=\;\frac{g_{z}+g_{y}}{D};\;b\;=\;\frac{g_{z}-g_{x}}{D};\;c\;=\;\frac{g_{y}-g_{x}}{D};$$

With $D\;=\;{\left[8\left(g_{z}+\;g_{y}-\;g_{x}\right)\right]}^{1/2}$ The ratio of the crystal field parameters and the spin-orbit coupling constant, *V*/*ξ* and Δ/ξ are then calculated from the *g*-tensor values according to the following equations:

(5) $$\frac{V}{\xi }=\frac{g_{x}}{g_{z}-\;g_{y}}+\frac{g_{y}}{g_{y}-\;g_{x}}$$

(6) $$\frac{\Delta }{\xi }=\frac{g_{x}}{g_{z}-\;g_{y}}+\frac{g_{z}}{g_{y}-\;g_{x}}-\frac{V}{2\xi }$$

Note that the knowledge of all three *g*-values is necessary in order to determine *V/ξ* and *Δ/ξ*. Also, the spin orbit coupling constant of free iron is *ξ*∼ 400 cm^−1^.

If this model is an accurate description for the iron centre, $a^{2}+b^{2}+c^{2}\equiv 1$. That is, all these equations are valid as long as the *t_2g_* orbitals are purely non-bonding and the remaining two empty *e_g_* orbitals (|dx^2^-y^2^〉 and |dz^2^〉) lie sufficiently high in energy that their contributions can be neglected. If we define the normalization parameter as:

(7) $$m^{2}=\;a^{2}+b^{2}+c^{2}$$

It can provide an idea of the degree of suitability of the analysis. If the value is very close to one, the description will prove valid. On the other hand, if the value is less than one, it has been interpreted as an indication for the existence of some degree of covalency, or delocalization of the spin density onto the ligands [5]. Conversely, if *m* is larger than one, it can be an indication of mixing of excited states with high orbital contributions (i.e. excited orbitals perturbing the ground state) [6,7] Table 2.

**Table 2:** Parameters calculated from the crystal field analysis of CYP116B5hd as well as of the reference protein CYPBM3hd.

		*g_z_*	*g_y_*	*g_x_*	*V*/*ξ*	Δ/*ξ*	*V/Δ*	*a*	*b*	*c*	*m^2^*
CYP116B5hd		2.440 ± 0.005	2.25 ± 0.002	1.92 ± 0.002	4.74 ± 0.06	5.44 ± 0.18	0.87 ± 0.32	0.996	0.110	0.085	1.012
CYP116B5hd + Imidazole	(1)	2.585 ± 0.005	2.26 ± 0.002	1.90 ± 0.002	4.42 ± 0.05	5.14 ± 0.17	0.86 ± 0.28	0.994	0.118	0.068	1.007
	(2)	2.468 ± 0.005	2.26 ± 0.002	1.86 ± 0.002	3.51 ± 0.03	5.12 ± 0.17	0.69 ± 0.16	0.991	0.148	0.068	1.009
CYP116B5hd + Histidine		4.419 ± 0.005	2.24 ± 0.002	1.92 ± 0.002	4.89 ± 0.06	5.48 ± 0.18	0.89 ± 0.34	0.995	0.107	0.069	1.006
CYPBM3hd		2.418 ± 0.005	2.26 ± 0.002	1.92 ± 0.002	4.98 ± 0.06	5.16 ± 0.17	0.97 ± 0.37	0.997	0.105	0.071	1.009
CYPBM3hd + Imidazole	(1)	2.450 ± 0.005	2.26 ± 0.002	1.90 ± 0.002	4.53 ± 0.05	4.98 ± 0.17	0.91± 0.30	0.994	0.116	0.076	1.007
	(2)	2.560 ± 0.005	2.26 ± 0.002	1.86 ± 0.002	3.64 ± 0.03	5.05 ± 0.17	0.72 ± 0.18	0.991	0.143	0.081	1.010
	(3)	2.610 ± 0.005	2.26 ± 0.002	1.85 ± 0.002	3.34 ± 0.02	5.01 ± 0.17	0.67 ± 0.14	0.990	0.155	0.084	1.012

It is worth noting that the calculated values for *m* are very close to 1 in all cases, which indicates the suitability of the analysis presented in Table 2 and, therefore, a predominant non-bonding character of the orbital hosting the unpaired electron with negligible admixture of higher energy terms.

## Ethics Statements

The work did not involve any human or animal subjects, nor data from social media platforms*.*

## CRediT authorship contribution statement

**Antonino Famulari:** Investigation, Validation, Formal analysis, Writing – original draft, Data curation. **Danilo Correddu:** Investigation, Resources, Validation. **Giovanna Di Nardo:** Conceptualization, Resources, Writing – review & editing. **Gianfranco Gilardi:** Conceptualization, Writing – review & editing. **Mario Chiesa:** Conceptualization, Supervision, Writing – review & editing, Funding acquisition. **Inés García-Rubio:** Conceptualization, Investigation, Supervision, Writing – original draft, Funding acquisition.

## Declaration of Competing Interest

The authors declare that they have no known competing financial interests or personal relationships that could have appeared to influence the work reported in this paper.

## Data Availability

CW-EPR, HYSCORE and simulations (Original data) (Zenodo).

## References

[1] Famulari A., Correddu D., Di Nardo G., Gilardi G., Chiesa M., García-Rubio I. (2022). EPR characterization of the heme domain of a self-sufficient cytochrome P450 (CYP116B5). J. Inorg. Biochem..

[2] Alonso P.J., Martínez J.I., García-Rubio I. (2007). The study of the ground state Kramers doublet of low-spin heminic system revisited. A comprehensive description of the EPR and Mössbauer spectra. Coord. Chem. Rev..

[3] Zoppellaro G., Bren K.L., Ensign A.A., Harbitz E., Kaur R., Hersleth H.P., Ryde U., Hederstedt L., Andersson K.K. (2009). Studies of ferric heme proteins with highly anisotropic/highly axial low spin (S = 1/2) electron paramagnetic resonance signals with bis-histidine and histidine-methionine axial iron coordination. Biopolym. Pept. Sci. Sect..

[4] Griffith J.S. (1956). On the magnetic properties of some haemoglobin complexes. Proc. R. Soc. Lond. Ser. A. Math. Phys. Sci..

[5] Taylor C.P.S. (1977). The EPR of low spin heme complexes Relation of the t2g hole model to the directional properties of the g tensor, and a new method for calculating the ligand field parameters. BBA Protein Struct..

[6] Griffith J.S. (1971). Theory of E.P.R. in low-spin ferric haemoproteins. Mol. Phys..

[7] Harris S. (1968). Convexity of the H function: The weak-coupled master equation. J. Chem. Phys..

[8] Ciaramella A., Catucci G., Di Nardo G., Sadeghi S.J., Gilardi G. (2020). Peroxide-driven catalysis of the heme domain of A. Radioresistens cytochrome P450 116B5 for sustainable aromatic rings oxidation and drug metabolites production. N. Biotechnol..

[9] Omura T., Sato R. (1964). The carbon monoxide-binding pigment of liver microsomes. I evidence for its hemoprotein nature. J. Biol. Chem..

[10] Stoll S., Schweiger A. (2006). EasySpin, a comprehensive software package for spectral simulation and analysis in EPR. J. Magn. Reson..

[11] Höfer P., Grupp A., Nebenführ H., Mehring M. (1986). Hyperfine sublevel correlation (HYSCORE) spectroscopy: a 2D ESR investigation of the squaric acid radical. Chem. Phys. Lett..

